# Green synthesis of magnesium nanoparticles mediated from *Rosa floribunda* charisma extract and its antioxidant, antiaging and antibiofilm activities

**DOI:** 10.1038/s41598-021-96377-6

**Published:** 2021-08-19

**Authors:** Inas Y. Younis, Seham S. El-Hawary, Omayma A. Eldahshan, Marwa M. Abdel-Aziz, Zeinab Y. Ali

**Affiliations:** 1 Department of Pharmacognosy, Faculty of Pharmacy, Cairo University, El Kaser El-Aini, Cairo, 11562 Egypt; 2 Pharmacognosy Department, Faculty of Pharmacy, Ain Shams University, Cairo, Egypt; 3 Medical Microbiology, The Regional Center for Mycology and Biotechnology (RCMB), Al-Azhar University, Cairo, Egypt; 4 Department of Biochemistry, Egyptian Drug Authority (EDA), Giza, 12553 Egypt

**Keywords:** Plant sciences, Medical research, Nanoscience and technology

## Abstract

Flower based nanoparticles has gained a special attention as a new sustainable eco-friendly avenue. *Rosa floribunda charisma* belongs to modern roses with bright yellow, red flowers with marvellous rose scent. Different methods were used for the extraction of its floral scent such as hexane, microwave, and solid-phase micro-extraction. The latter was the most efficient method for the extraction of phenyl ethyl alcohol, the unique scent of roses. In the current study, magnesium nanoparticles (RcNps) have been synthesized using *Rosa floribunda* charisma petals that have privileges beyond chemical and physical routs. RcNps formation was confirmed using UV–Visible (UV–Vis) Spectroscopy, Fourier Transform Infrared Spectroscopy (FTIR), High Resolution-Transmission Electron Microscope (HR-TEM), Field Emission-Scanning Electron Microscope (FE-SEM), Energy dispersive X-ray (EDX), X-ray Diffractometer (XRD), and X-ray photoelectron spectroscopy (XPS). HR-TEM images detected the polyhedral shape of RcNps with a diverse size ranged within 35.25–55.14 nm. The resulting RcNps exhibited a high radical scavenging activity illustrated by inhibition of superoxide, nitric oxide, hydroxyl radical and xanthine oxidase by by IC_50_ values 26.2, 52.9, 31.9 and 15.9 µg/ml respectively as compared to ascorbic acid. Furthermore, RcNps at concentration of 100 µg/ml significantly reduced xanthine oxidase activity (15.9 ± 0.61 µg/ml) compared with ascorbic acid (12.80 ± 0.32 µg/ml) with *p* < 0.05. Moreover, RcNps showed an excellent antiaging activity demonstrated by inhibition of collagenase, elastase, hyaluronidase and tyrosinase enzymes in a dose-dependent manner with IC_50_ values of 58.7 ± 1.66 µg/ml, 82.5 ± 2.93 µg/ml, 191.4 ± 5.68 µg/ml and 158.6 ± 5.20 µg/ml as compared to EGCG respectively. RcNps also, exhibited a promising antibacterial activity against three skin pathogens delineate a significant threat to a public health, as *Staphylococcus epidermidis*, *Streptococcus pyogenes*, and *Pseudomonas aeruginosa* with MIC of 15.63, 7.81, 31.25 µg/ml as compared to ciprofloxacin (7.81, 3.9 and 15.63 µg/ml). Moreover, RcNps suppressed the formation of biofilms with minimum biofilm inhibitory concentrations 1.95, 1.95, 7.81 µg/ml against the fore mentioned strains, respectively. Overall, our findings indicate that *Rosa floribunda* nanoparticles could be used as a leading natural source in skin care cosmetic industry.

## Introduction

Green synthesis has gained a great attention as a sustainable, reliable, and eco-friendly approach for the synthesis of a variety of nanomaterials. By twenty-first century, it is described as the wonder of medicine that leads to industrial revolution^1^. Among the different approaches in skin rejuvenation, the use of nanoparticles(Nps) loaded with cosmeceuticals (e.g., phytochemicals, vitamins, and hyaluronic acid) has become an interesting alternative^2^ . Importanly, plant based Nps are a valuable source of myriad bioactive metablolites as ascorbic acid , flavonoids, alkaloids and terpenoids. They surve as a natural bio-reductaned of metal ions as wellas as capping agents for sterically stabilizing Nps through the reduction of the direct interaction between molecules^3,4^.

The revolution of metal-based nanoparticles represents an important breakthrough in nanotechnology towards the production of high-quality products. A scientific survey indicates that biowaste from fruits and vegetables peel rich in vast array of biomolecules are dumped in huge quantities without management which ultimately leads to the generation of unhygienic condition for the market vendors^4^.The sustainable solutions for waste reduction can be established through its utilization for the production of valuable nano particles .

Among the the most important metal based Nps synthesized via the green techniques are iron, zinc , copper , gold, silver and their oxides^5^ . Currently , *Artocarpus heterophyllus* peel has been successfully used for the preparation of iron nanoparticles^4^. Similarly, *Lansium domesticum* and *Phyllanthus emblica* fruits can synthesize gold nanoparticles with an efficient antimicrobial activity^6^. The extensive interest of Nps can be attributed to their tailorable physicochemical properties and wide rang of applications in the pharmaceutical and industrial fields.

In the industrial field ,carbon nanostructures have been recently established for the production of an expectionally photocatalytic activity and an outstanding electrical conductivity in the field of green fuels, water purification , and energy storage devices^7,8^.

In the pharmaceutical field, silver nanoparticles have been intensively studied due to their unique physicochemical properties that utilized in many cosmetics preparations with a broad range of antibacterial activity^6^. Rapaid synthesis of AgNps also has been achieved using *Calendula officinalis* and *Capsicum annuum* L.seeds extracts^6^. Currently, *Camellia sinensis* leaves are used in the extracellular synthesis of ZnONps as a catalytic agent^6^.

Despite of the wide application of Ag and ZnO nanoparticles, they are usually associated with high risk of toxicity due to their accumulation in the body. In contrast, magnesium is an important component required for the growth of plant . It acts as a powerhouse in the the photosynthesis process. Moreover, it shows a potent interaction with plant phytoconsituents to yield Nps. Magnesium oxide nanoparticles (MgONps) serve as a safe alternative with an extremely effective antibacterial activities as recognized by FDA^5^. They have been used as a superior nanocarrier with unique biocompatible nature and stable physicochemical properties. They have the advantage of being highly ionic with photocatalytic characteristics and an effecient tolerance to high temperature. Recently, they have been employed as a noval application in the refractory material and as a substrate in the biomedical field. Despite of their promising antimicrobial activity against *Staphylococcus aureus*, *Bacillus subtilis,and Pseudomonas aeruginosa*^9^*.* But, a little is known about their antioxidant, antiaging and antibiofilm activities in dermatological formulations.

Nature is an inexhaustible source of wonderful flowers where, rose is undeniably described as the “Queen of Flowers” and has been honored as a symbol of beauty, serenity, and innocence^10^. Today, roses have acquired a cultural importance in many societies not only for their unique floral scent but also for their vast array of health benefits as antibacterial, analgesic, and antidiabetic activities^11^. Hitherto, more than 1000 genotypes of roses are discovered; but still a few of them is preferred in the manufacture of perfumers as *Rosa damascene* Mill., *Rosa alba* L., and *R. gallica* L^10^.

Unlike the ancient species of roses that bloom once a year, modern roses continue blooming with beautiful large flowers. *Rosa floribunda charisma* is a modern group of garden roses results from crossbreeding of tea roses with polyantha roses. It is characterized by bright attractive yellow, red flowers with marvelous rose scent and long vase life^12^.

The chemical profile of the floral scent is greatly affected by the extraction method. Historically, several methods were established to capture the volatile scent including the traditional methods as hexane, steam distillation along with the advanced techniques as solid phase microextraction, matrix solid-phase dispersion and microwave-assisted extraction methods^13,14^. Although the extraction process may sound only to be of a great interest for the organoleptic property of the oil but, it is also considered as the key step that influences the quality of the oil^14^ .

Rose oil is established as one of the most beautiful and exquisite in the world. More than 300 volatile compounds have been identified in rose oil in which citronellol ester and phenyl ethyl alcohol are the major components of the blooming *Rose damascene* Mill in addition to nonvolatile compounds as flavonoids and tannins with anti-collagenase activity^11^.

Skin aging is a dynamic process that involves structural alterations of collagen rich extracellular matrix (ECM)in unpredictable sequence^15^. A dramatic breakdown of long-life proteins such as elastin, collagen and fibronectin is the main hallmark that contributes to premature aging with continuous degradation of hyaluronic acid^16, 17^ .

Recently, reactive oxygen species (ROS) are reported as the main extrinsic factor participating in the natural skin aging. Additionally, ROS promote the chain reactions that ultimately, lead to a functional damage of the dermal ECM^18^.

*Staphylococcus epidermidis* is described as a part of the skin innate immunity that preserves its normal ecosystem. However,under specific conditions as skin flaking it becomes an opportunistic pathogen^19^ . While , S. *pyogenes* is facultative, *Gram*-positive cocci responsible for life-threatening human infections^20^ .On the other hand, *Pseudomonas aeruginosa* is an ubiquitous and opportunistic *Gram*-negative bacterium capable of causing ocular keratitis and wound infections^21^. Most of these bacteria exerted their virulence via biofilm formation^20–23^.

We report for the first time, the formulation of *Rosa floribunda* as MgONps and the investigation of their antioxidant, antiaging as well as, their antibacterial and antibiofilm activities against skin born pathogens. Specifically, our objective was to provide a deep insight into a new natural antioxidant-based nanoparticle as carrier of rose essential oil and to characterize their biological activites as skincare platform.

## Results and discussion

### Characterization of the volatile aroma by different methods

The floral scents of roses are complex mixtures of vast array of chemicals. However, alcohols, monoterpenes, esters, aldehyde, and hydrocarbons remain the most characteristic groups for each species. Even within species, there is a unique aroma for each cultivar. Volatile aroma can be extracted by different traditional and innovative techniques. The extraction method plays a crucial role in the chemical profile of the floral scent^11^.

Unfortunately, the traditional methods such as steam distillation and hydro-distillation are often used but they usually lead to the degradation of thermo-labile compounds^24^ . In this study, a great fluctuation was observed in the volatile profile with respect to extraction method. Hexane extraction represents the classical method of extraction in parallel with two innovative techniques as microwave assisted extraction (MEA) and head space solid phase microextraction (HS-SPME).

A total of 18 components were identified by three different extraction techniques as illustrated in Table 1 and Supp. 1. Among these compounds, hydrocarbons were the most abundant volatile compounds in hexane extraction > SPME > MAE accounting for 64.54%, 46.13%, 4.43% respectively (calculated as % peak area of GC–FID analysis). Heptacosane and nonacosane were the major long-chain hydrocarbons extracted by this method. High ratio of alkanes and alkenes in the volatile aroma of *R. damascene* had been previously reported^13^ . Recently, several hydrocarbons as heptadecane and nonadecane have been detected in several *Rosa* taxa as *R. damascene* Mill, *R. alba* L. and *R. gallica*^10^ . Although hydrocarbons do not participate to the volatile scent of roses, but they contribute to the stability of the aroma^11^.

**Table 1:** Identification of the key aroma components of *R. floribunda* by hexane extraction, microwave assisted extraction, solid phase micro-extraction.

Component	Molecular formula	%Composition of hexane extract	%Composition of MAE	Composition % of SPME/GC	KI
**Hydrocarbons**					
*n*-Heneicosane	C_21_H_44_	–	4.43	–	2096^a^
*n*-Pentacosane	C_25_H_52_	3.41	–	–	2495^a^
*n*-Heptacosane	C_27_H_56_	27.65	–	–	2696^a^
1,6-Heptadyne	C_7_H_10_	–	–	12.17	1039 ^b^
Di-cycloheptadiene	C_10_H_12_	–	–	1.32	1042 ^b^
Cyclobutene, 2-propenylidene	C_7_H_8_	–	–	32.64	1114^b^
n-Octacosane	C_28_H_58_	2.08	–	–	2789^a^
2-Methyloctacosane	C_29_H_60_	4.79	–	–	2835^a^
*n*-Nonacosane	C_29_H_60_	24.35	–	–	2891^a^
Triacontane	C_30_H_62_	2.26	–	–	3009^a^
Total hydrocarbons		64.54	4.43	46.13	
**Alcohols**					
Phenyl ethyl alcohol	C_8_H_10_O	7.62	14.64	35.75	1115^a^ 1113^b^
Total alcohols		7.62	14.64	35.75	
**Aldehyde**					
Furfural	C_5_H_4_O_2_	–	6.98	–	826^a^
Phenyl acetaldehyde	C_8_H_8_O	–	25.71	–	1042^a^
2-Butanal	C_4_H_6_O	–	–	5.73	1406^b^
Total aldehyde		0	32.69	5.73	
**Ketones**					
3- Methyl-4-heptanone	C_8_H_16_O	3.08	–	–	918^b^
Total ketones		3.08	0	0	
**Esters**					
Oxalic acid, allyl pentyl ester	C_7_H_10_O_4_	–	–	0.67	1086^b^
Oxalic acid, allyl ethyl ester	C_7_H_10_O_4_	–	–	11.49	1418^b^
Total esters		0	0	12.16	
**Phenols**					
Butylated hydroxytoluene	C_15_H_24_O	–	43.79	–	1515^a^
Total phenols		0	43.79	0	
Total identified compounds		75.24	95.55	99.77	

*MAE* microwave assisted extraction, *SPME* solid phase micro-extraction.

^a^KI (kovatis indices) on HP‐5 capillary column.

^b^KI (kovatis indices) on DB5-MS column.

Green technology utilizing HS-SPME represents a sensitive and a robust method to capture the unique floral scent of roses. SPME fiber was able to pre-concentrate the floral scent of phenyl ethyl alcohol (35.75%), prior to GC/MS more than two-fold the content recovered by MEA (14.64%) and fivefold the level of hexane extraction (7.62%). In a good agreement with previous studies, phenyl ethyl alcohol at different concentrations was the unique marker of several rose varieties as *R. damascene* Mill., *R. gallica* L., *R. moschata* Herrm. and *R. centifolia* L^25–27^ .Variation in the level of phenyl ethyl alcohol is characteristic for each type of roses depending on several factors as; environmental condition, blooming stage, harvesting time^11^.

In the same milieu, MAE is an innovative technique that successfully employed to increase extraction yield as compared to other conventional extraction methods. Phenyl acetaldehyde with its honey-like odor was identified only by MAE in high concentration (25.71%) accounting for one fourth of total content.

### Spectrophotometric characterization of RcNps

The MgONps were synthesized, for the first time, using the *Rosa floribunda* charisma (RcNps). Recently, several studies reported various biological methods for the synthesis of MgONps as a promising alternative to the traditional chemical methods, including the use of microorganisms like endobacterium *Burkholderia rinojensis*^28^ or plant extracts such as *Manihot esculenta*^29^ and *Pterocarpus marsupium* rox.b^30^*.*

**Figure 1:**
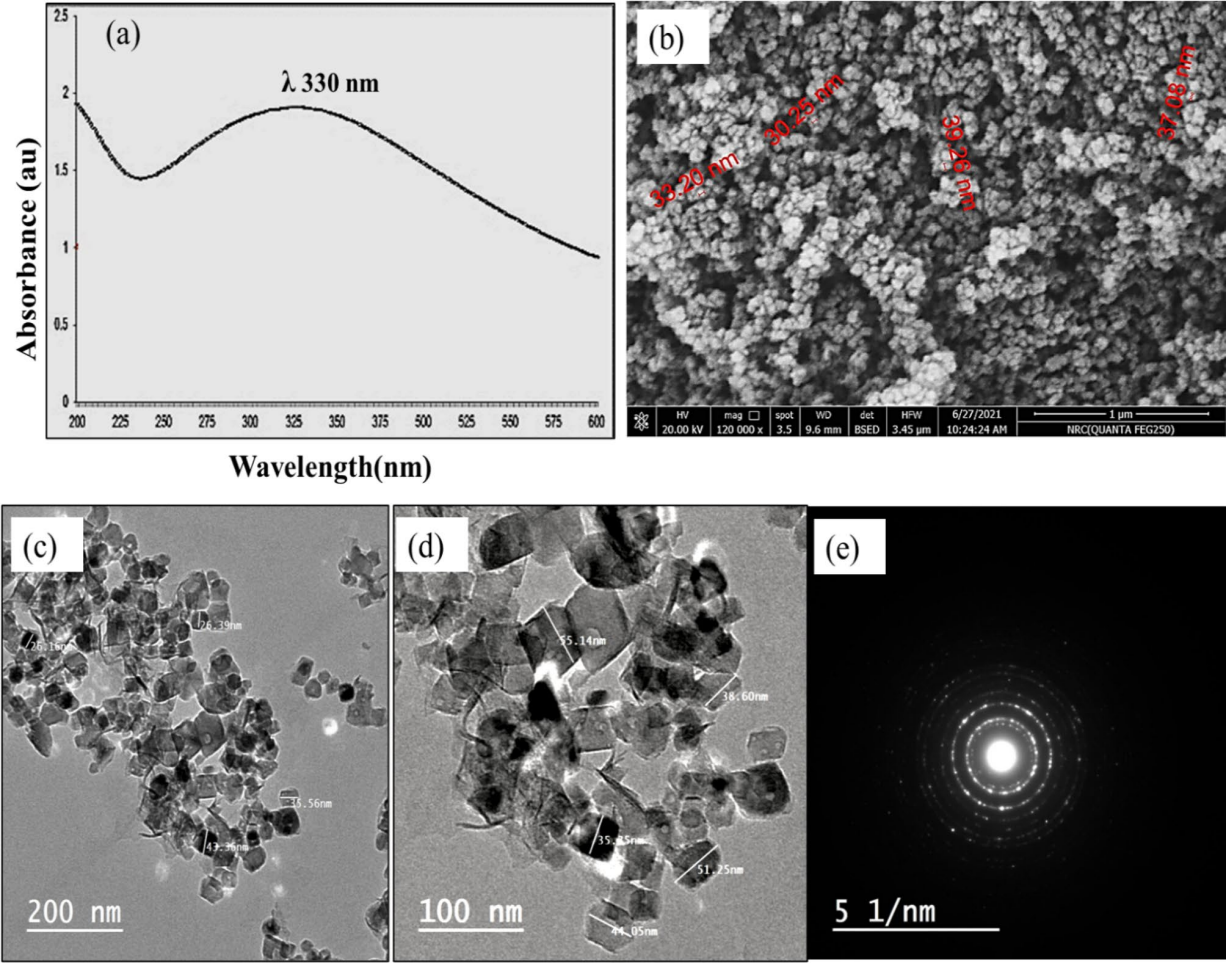
**(a)** UV–Visible absorption spectrum of RcNps. **(b)** FESEM micrograph of RcNps **(c)** HR-TEM micrograph of RcNps at 200 nm, **(d)** HR-TEM micrograph of RcNps at 100 nm, and **(e)** selected area for electron diffraction patterns showing the crystallinity of RcNps.

Size as well as surface properties of nanoparticles are an important criterion to determine their pharmacokinetic, bioavailability and biological activity^25^ .The optical properties of RcNps were detected in the wavelength range of 200–600 nm by UV–Vis analysis (Fig. 1a). In the UV–visible spectroscopy, the sharp absorbance peak at 330 nm indicated the formation of small sized particles of MgO according to Jeevanandam et al., 2017^31^ .The green synthesized Nps usually demonstrates a surface plasmon resonance, which leads to absorption in the UV–Vis region with distinctive optoelectronic properties^6^ .

The FESEM micrograph (Fig. 1b) showed agglomeration of RcNps, the agglomeration might be due to the electrostatic attraction of MgONps as reported by Pugazhendhi et al. 2018^32^. On the other hand, HR-TEM micrograph of RcNps (Fig. 1c–e) points to the polyhydral shape of the synthesieg RcNps, at different magnification power . In a good agreement with previously observed polyhydral structure of MgONps by Nguyen et al., 2018^33^. A close look at the RcNps using HR-TEM (at 100 nm) confirms the nanometer size with size ranges from 35.25 to 55.14 nm (Fig. 1d). The selected area for electron diffraction patterns show rings corresponding to the crystal planes of RcNps (Fig. 1e).

**Figure 2:**
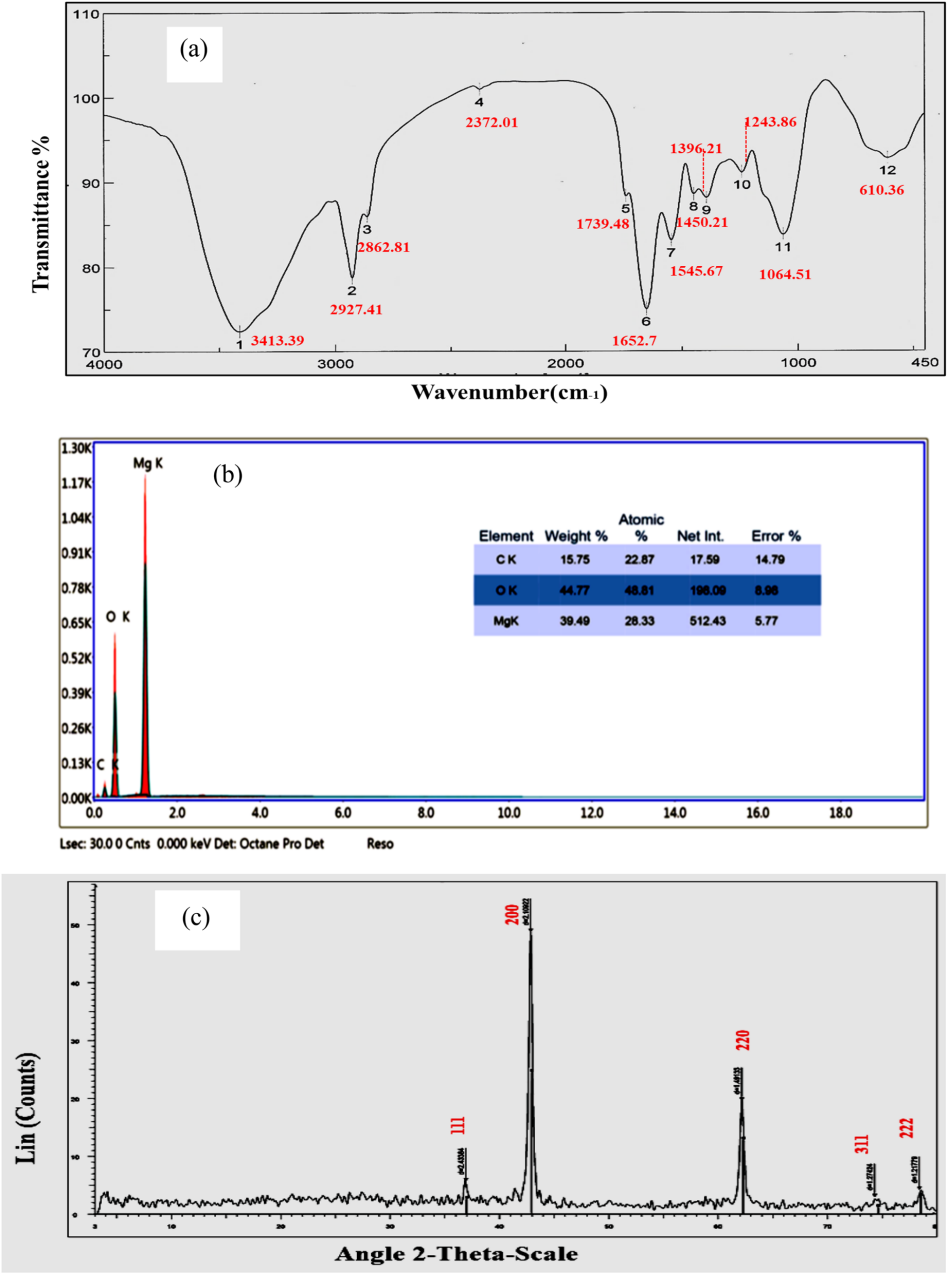
**(a)** FTIR spectrum of RcNps **(b)** EDX spectrum of of RcNps **(c)** XRD pattern of RcNps.

In the frame of the current study, the FTIR spectra (Fig. 2a) shows bands at 3308, 2139, 1635, 1346, 419 and 407 cm^−1^. The strong band near 3308 cm^−1^ was observed for O–H bond vibration of hydroxy group which was attributed to the presence of many bioactive metabolites as flavonoid , anthocyanin and tannins^11^.

The EDX spectrum (Fig. 2b) indicates the strong magnesium signal (with mean 39.49%)), and a peak from oxygen (with mean 44.77%) . The presence of a signal peak for carbon (with mean 15.75%) may be due to the bioactive metabolites of *Rosa floribunda* which play a dual role in both reducing and stabilizing RcNps^28^. Infact, high concentration of flavonoids markedly affect the formation of Nps^31^ .

The crystal structure and purity of the biologically synthesized RcNps have been determined by X-ray diffraction technique (XRD). The diffraction peaks in (Fig. 2c) highlighted the crystalline nature of the synthesized RcNps .The five distinct diffraction peaks at 36.902, 62.198, 62.198, 74.388, and 78.476 were corresponding to the planes (111), (200), and (220), (311), and (222), respectively. All of the diffraction peaks were readily indexed to various crystal planes of the cubic phase MgO and no secondary peaks can be detected, which indicates the purity of the sample^34^ .

**Figure 3:**
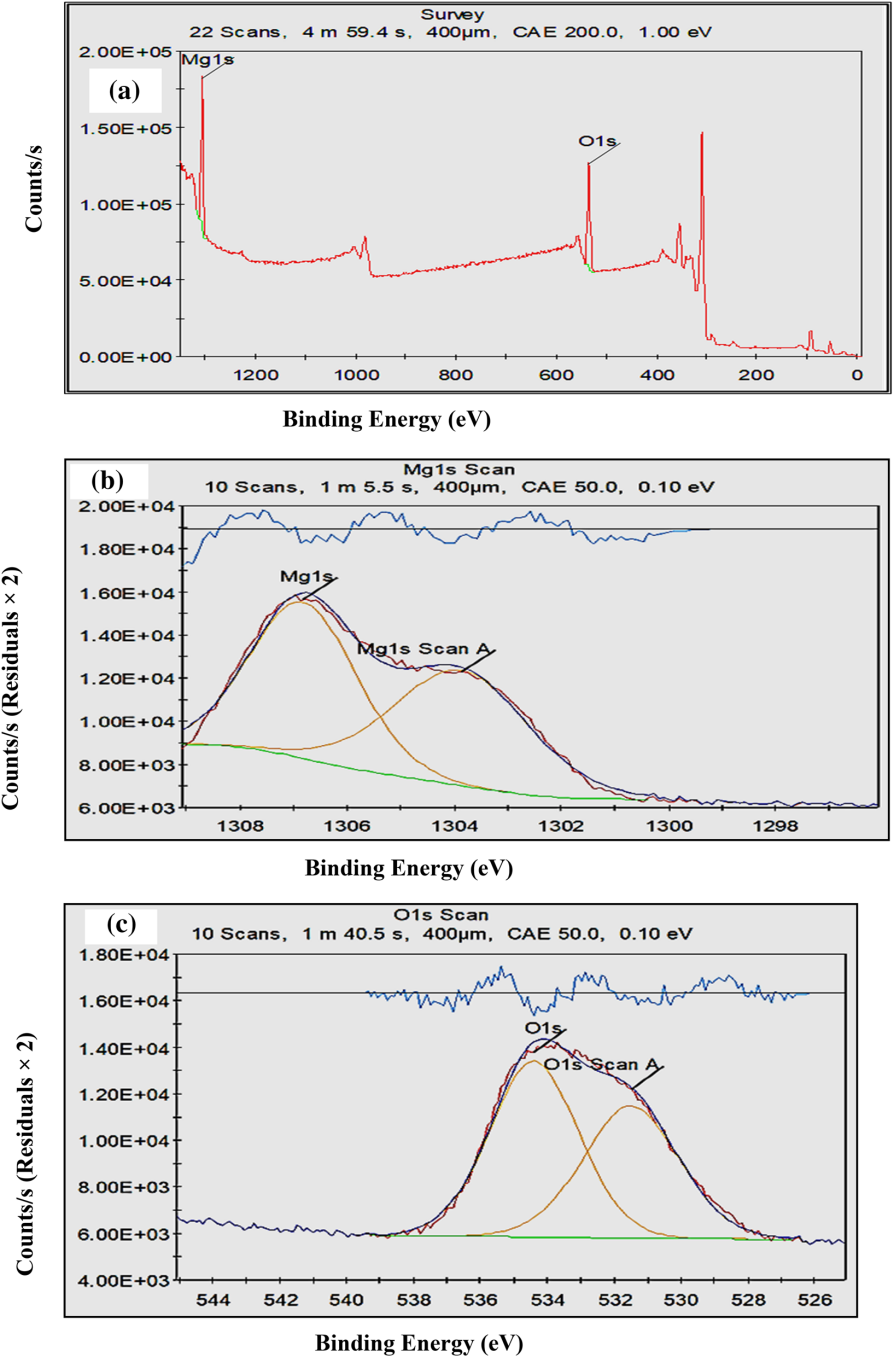
**(a)** XPS survey of RcNps as-synthesized MgO nanoparticles showing peaks for the elemental contents in the synthesized sample, **(b)** XPS pattern of RcNps, curve fitting of Mgs1 and **(c)** XPS pattern of RcNps, curve fitting of Os1.

To investigate the surface chemical features of RcNps, the sample was further characterized by X-ray photoelectron spectroscopy (XPS). The XPS survey spectra (Fig. 3a) revealed two peaks at 534.69 eV(labbled as O1s) and 1305.69 eV(labbleb as Mg1s). Furthermore, Fig. 3b,c revealed the deconvoluted Mg1s and O1s spectra. The XPS survey data (Table 2) represented for O1s, and Mg1s, indicating the existence of Mg and O elements, further confirm the purity of RcNps. The survey spectrum matched with the previously reported study^34^ .

**Table 2:** XPS data of biosynthesized RcNps.

Name	Peak BE	FWHM eV	Area (P) CPS.eV	Atomic %	Q
Mg1s	1305.69	5.35	551,907.5	46.83	1
O1s	534.69	6.31	444,589	53.17	1

### In-vitro assessment of RcNps as antioxidant

Skin aging is a complex progressive process that involves oxidative structural and physiological damage of skin with gradual loss of its elasticity^11^ . One of the most widely accepted theory of aging, free radical theory of aging (FRTA), proposes that oxidative damage caused by ROS is the primary cause of aging . FRTA assumes that aging is driven by the accumulation of ROS-induced macromolecular damage^35^ . Several lines of evidence confirm that oxidative stress was directly involved in skin premature aging along with UV irradiation^36,37^.

Dietary supplementation with natural antioxidants plays a crucial role in delaying aging-associated pathological conditions through their interaction with free radicals, terminating the adverse chain reactions and converting them to harmless products^35,36^. However, the therapeutic potential of natural antioxidant was restricted due to low solubility, stability, difficulties to cross the cell membranes, and hence poor bioavailability. Recently, the delivery of natural antioxidants based Nps showed successive improvements through an innovative green nanoscience^38^. Consequently, it is quite interesting to investigate the effect of the nano form of *Rosa floribunda* (RcNps) as natural antioxidants to protect skin from the oxidative damage of ROS.

**Figure 4:**
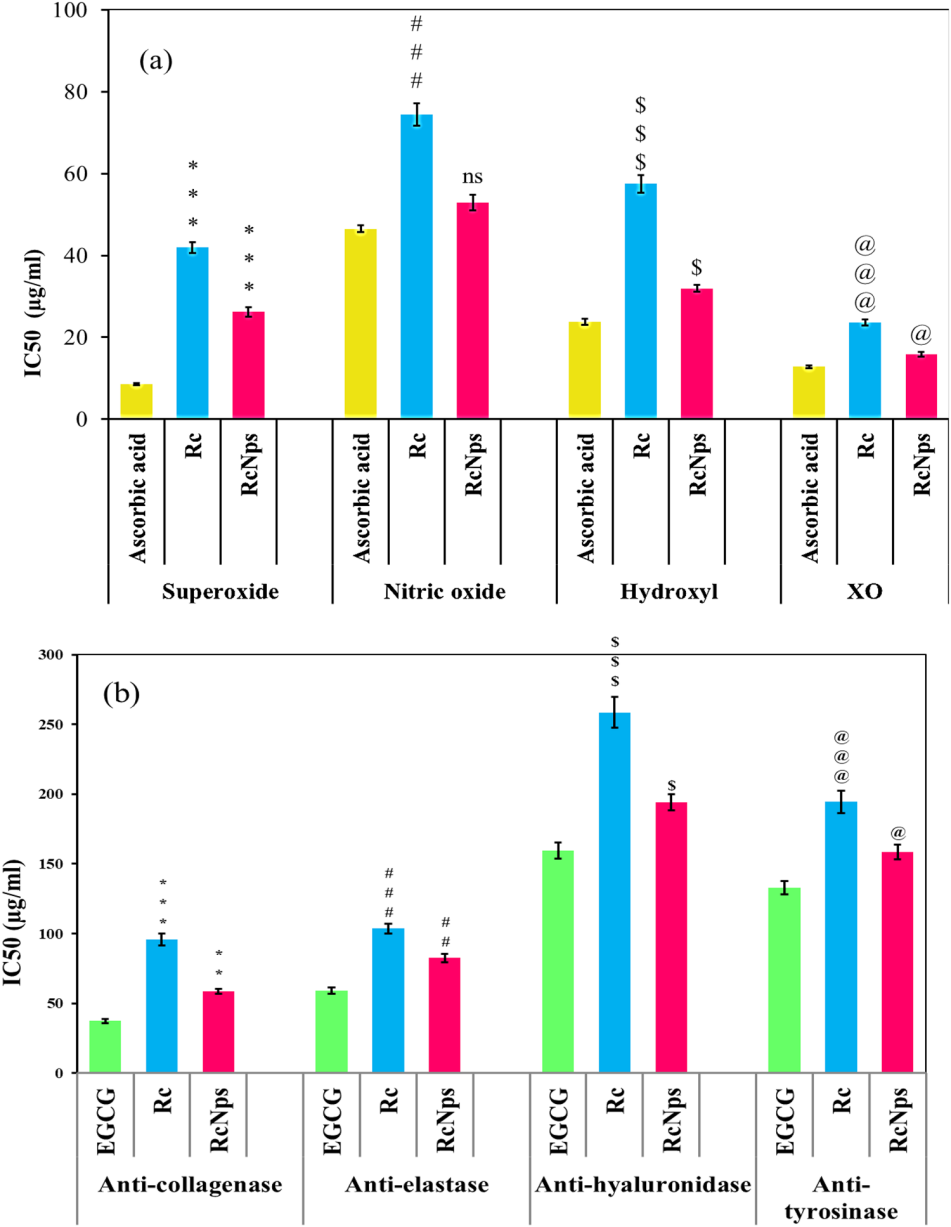
**(a)** Antiaging activity of the extract of *Rosa* (Rc) and its nano-form (RcNps) expressed as IC_50_ in comparable to reference ascorbic acid. **(b)** Antiaging activity of the extract of *Rosa* (Rc) and its nano-form (RcNps) expressed as IC_50_ in comparable to epigallocatechin-3-gallate(EGCG) .The data expressed as the mean of three parallel measurements (n = 3). One-way ANOVA followed by Dunnett’s multiple comparisons test statistically tested the differences between means. Significant difference from corresponding RA values was tested at @P < 0.05, **, ##P < 0.01 and ***, ###, $$$, @@@P < 0.001.

The antioxidant activity was assessing the ability of RcNps to neutralize the free radicles using standard methods . The concentration of the sample in µg/ml required to exhibit 50% effect (IC_50_) is inversely related to the activity. In the present study, the radical scavenging potential was found to be decreased in the order of superoxide anions > hydroxyl > nitric oxide by IC_50_ values of 26.2, 31.9, and 52.9 μg ml^−1^, respectively for RcNps, and 42.0, 57.5, and 74.5 μg ml^−1^, respectively for Rc compared to ascorbic acid as a reference standard of antioxidant (Fig. 4a).

Our results demonstrated that both original Rc and its nano form RcNps exhibited an effective dose-dependent antioxidant activity. This beneficial activity is more pronounced in RcNp (Supp. 2.). This antioxidant activity may be mediated by two different mechanisms involved radical scavenging potential and inhibition of xanthine oxidase. The antioxidant activity of Rc is believed to be mainly due to redox properties of its active constituents mainly quercetin glycosides and gallic acid derivatives with different potency. They are playing an important role in adsorbing and neutralizing free radicals^11,39^ .Chemically, the unique features of quercetin represented by O-dihydroxyl groups of catechol B-ring with additional three hydroxyls at 3, 5 and 7positions enhance the anti-oxidative property^36^.

Xanthine oxidase, one of the most important ROS-generating enzymes, produces superoxide anions during the break down of purines to uric acid. The obtained results reveal that RcNps exhibited a potent inhibitory effect on XO with an IC_50_ value of 15.9 μg ml^−1^ than that of Rc (23.6 μg ml^−1^) as compared with ascorbic acid (12.8 μg ml^−1^).

These results indicate that the nano form (RcNps) led to a considerable improvement of the antioxidant activity. The pronounced antioxidant activity of RcNps increases its therapeutic value for free radical-mediated diseases such as aging. In consistent with our results, Manikandan et al., 2015^40^reported the potential antioxidant activity of silver nanoparticles loaded with *Rosa indica* against the generation of superoxide anion and nitric oxide in rat macrophages. Furthermore, MgONps loaded with *Artemisia abrotanum* extract exhibited a good antioxidant activity and a potent catalytic effect in the reduction of methyl orange^41^ .

Recently, Chahardoli et al., 2020^42^ reported a high antioxidant potential of aluminum and nickel nanoparticles of *Nigella arvensis* L. Yet another study, suggested that the volatile biomolecules of *Manihot esculenta* leaf extract act as natural bio-reluctant and capping agents that stabilize the synthesized MgONps^29^ . Utilizing DPPH radical scavenging method, it was reported that the synthesized MgONps enhanced the antioxidant activity of the *Manihot esculenta* herb extract^29^ and *Pterocarpus marsupium* rox.b^30^ .

### In-vitro evaluation of RcNps as anti-aging

Excessive exposure to ultraviolet radiation triggers the photochronical generation of ROS, that stimulates over expression of matrix metalloproteinases enzymes such as collagenase and elastase resulting in degradation of the extracellular matrix (ECM) especially collagen, and elastin^17^. These enzymes played fundamental roles in the proteolytic degradation of ECM required for skin rejuvenation^43^. In addition, the destructive damaging effects of ROS are usually associated with the enhancement the activity of tyrosinase and hyaluronidase enzymes. The enhancement of tyrosinase, a key enzyme in the melanin synthesis leading to atypical hyperpigmentation^27^. Meanwhile, stimulationof the hyaluronidase results in breakdown of hyaluronic acid and appearance of fine wrinkles^44^ .

Based on the traditional use of roses in several cosmetic formulation, the present study was established inorder to evaluate the inhibitory effect of RcNps and Rc at different concentrations on collagenase, elastase, hyaluronidase and tyrosinase as potential markers of anti-aging activity. The results indicated that both RcNps and Rc exhibited a dose dependent inhibition decreased in the order of collagenase > elastase > tyrosinase > hyaluronidase at IC_50_ values of 58.7, 82.5, 158.5 and 196.1 μg ml^−1^, respectively for RcNps, and 95.8, 103.7, 194.4 and 258.6 respectively for Rc as compared to EGCG standard (Fig. 4b, Supp. 3). RcNps exhibited higher catalytic activity against all prospective enzymes than Rc.

Plants are a rich source of bioactive phytochemicals associated with a reduced risk of many chronic diseases. Several studies have reported that the anti-aging activity of the plant has been attributed to their ability to reduce free radical damages to the skin, along with their capacity to modulate the activity of enzymes involved in aging process such as elastase, hyaluronidase, collagenase and tyrosinase^30,41^.

It was reported that the beautiful volatile scent of roses especially phenyl ethyl alcohol may be responsible for the anti-tyrosinase activity of *Rosa rugosa* Thunb by-product^27^ . In addition to other natural antioxidant.Quercetin, cyanidin along with their glycosides , gallic acid and its dimeric form ellagic acid are among the major bioactive metabolites that have been identified in several *rosa* taxa^11,25,27^. A substantial studies have emphasized their antiaging potential mediated through inhibition of the generation of ROS^36^.

In particular, quercetin Nps were more effective as antioxidant than pure quercetin^36^. Recently, Ammulu et al., 2021^30^ highlights the improvement of the catalytic activity of MgONps loaded with *Pterocarpus marsupium* rox.b heartwood extract . The present study revealed the anti-aging potential of *Rosa floribunda* plant extracts mediated through antioxidant and anti-enzyme activities.

### Minimum inhibitory concentration and minimum biofilm inhibitory concentrations

The green synthesis of plant-based Nps has gained more attention as a new promising revolutionary technology not only for its simplicity, and eco-friendly but also, as a safe alternative to synthetic antibiotics. Several researchers highlighted the discrete activity of Nps targeting multiple bacterial biomolecules such as ribosomes, DNA, and enzymes^45^. Nps can also interfere with the permeability of the cell membrane, inhibit the oxidative stress, and gene expression^5,45^.

**Figure 5:**
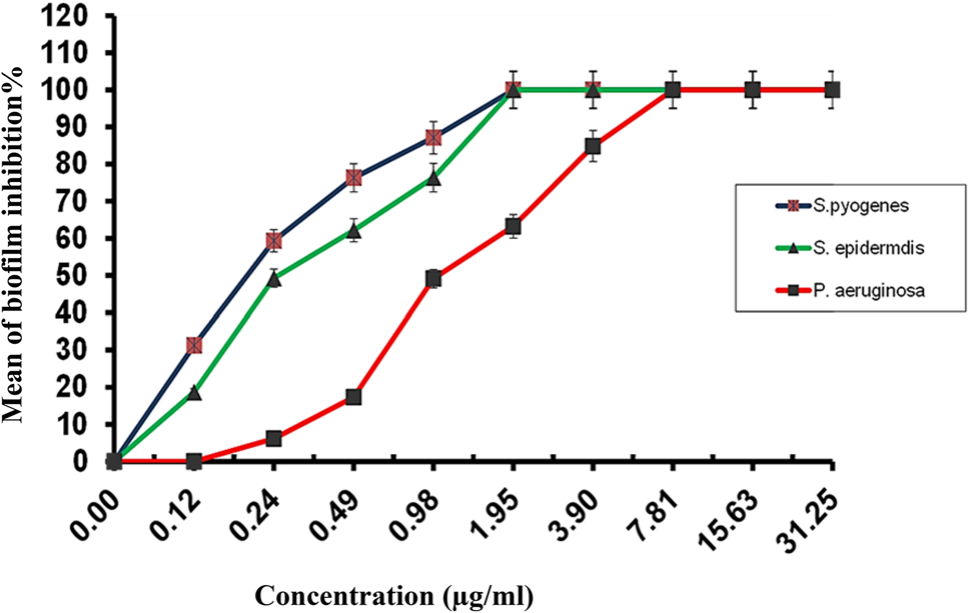
Mean of biofilm inhibitory percentages of RcNps against *Staphylococcus epidermidis*, *Streptococcus pyogenes*, and *Pseudomonas aeruginosa*.

In the current study, RcNps were active against *Staphylococcus epidermdis, Streptococcus pyogenes* and *Pseudomonas aeruginosa* in their planktonic form with MICs of 15.63, 7.81, 31.25 µg/ml as compared to ciprofloxacin which showed 7.81, 3.9 and 15.63 µg/ml against the fore mentioned strains, respectively. Concerning antibiofilm activity, RcNps were able to inhibit the biofilm formation of the three strains in a dose dependent manner (Fig. 5). RcNps, at concentration of 0.98 µg/ml, suppressed *Staphylococcus epidermdis*, *Streptococcus pyogenes* and *Pseudomonas aeruginosa* biofilms by 76.35, 87.15, and 49.14%, respectively, with minimum biofilm inhibitory concentrations (MBICs) of 1.95, 1.95, 7.81 µg/ml for the mentioned strains respectively.

In consistent with previously reported data Karunakaran et al., 2016^46^pointed to the potential antibacterial activity of MgONps loaded with *Hydrangea paniculata* flower over streptomycin against *E. coli* and *S. aureus*. Their potential activity was directly linked to their antioxidant capacity, which in turn inhibited bacterial enzymes.

Recently, a similar study was conducted by Abdallah et al., 2019^47^who confirmed the promising antibacterial activity of rosemary flowers MgONps through interfering with bacterial biofilm formation, and swimming motility.

## Material and methods

### Plant material

Fresh flowers of *Rosa floribunda* charisma ™ yellow, red (Charisma Rose) were collected from Orman Botanical Garden (Giza, Egypt) during March 2017after permission of Agriculture Research Center, Giza Egypt at "9 Cairo university Road, Giza District, Giza Governorate". The collection of plant material was established in compliance with the national guidelines. They were kindly authenticated by Professor Mohamed El-Gebaly (Department of Botany, National Research Centre (NRC)). A voucher specimen no. (25-3-17) was deposited the Herbarium of, Pharmacognosy Department, Faculty of Pharmacy, Cairo University. The flower petals were dried in shade then grinded in coffee grinder and kept for analysis. Two parts of dried powdered petals (each of 100 g) were extracted three times with 80% ethanol and n-hexane (3 × 500 ml). The pooled extracts were evaporated under reduced pressure at 40 °C until complete dryness and stored in brown color bottles at − 20 °C. The alcoholic extract was used for *in-vitro* assay of antioxidant and antiaging activities.

### Chemicals and reagents

All chemicals, terpenes standards as well as kits for antioxidant and antiaging activities were provided from Sigma Aldrich (St. Louis, Mo., U.S.A.). All reference standards as clarithromycin (≥ 95%, HPLC), l-ascorbic acid (≥ 99%) and (−) epigallocatechin gallate (≥ 95%) were purchased from Sigma Aldrich in addition to Mg (NO_3_)_2_.6H_2_O for synthesis of nanoparticles. SPME holder and fiber coated with 100 μm polydimethyl siloxane (PDMS) were purchased from Supelco (Oakville, ON, Canada).

### Bacterial strains

Three biofilm-positive American Type Culture Collection [ATCC] strains were used in this study namely, *Staphylococcus epidermdis* (ATCC 35984), *Streptococcus pyogenes* (ATCC 19615), *Pseudomonas aeruginosa* (ATCC 700829). Mueller Hinton Broth (MHB)was purchased from HiMedia Laboratories, India.

### Characterization of the volatile aroma by different methods

#### Preparation of n-hexane extract

The dried flowers were extracted with n-hexane. The pooled extracts were evaporated under reduced pressure at 40 °C until dryness. The characteristic volatile components were analyzes using the condition described in the next section.

### Microwave assisted extraction coupled with GC-FID/MS

Microwave Assisted Extraction (MEA) is an advanced technique that utilizing the power of microwave for an efficient extraction of the volatile constituents. The method of^48^ was applied with slight modifications, The CEM, MARS 6 Microwave Reaction System (CEM Corp., Mattews, NC, USA) with microwave power of 800 W at 100 °C was used for aqueous extraction of 100 g of dried petals for 60 min.

GC analysis of the volatile scent extracted by hexane extraction and MEA were conducted using the following conditions: GC HP 5890 Hewlett Packard (Agilent Technologies, Palo Alto, CA, USA) equipped with FID and HP‐5 fused silica capillary column (30 m × 0.25 mm i.d., film thickness 0.25 μm). Sample volume was set to be 0.03 μl and oven temperature was programmed from 60 °C to 240 °C at 3 °C/min. The injector and detector temperatures were maintained at 250 °C and 280 °C with a flow rate of helium as a carrier gas. The automatic sample injection was 0.02 μl of the oil with split of 1/70. The relative proportions of the essential oil constituents were expressed as percentages obtained by peak area normalization.

While, for detection of mass Perkin–Elmer quadrupole MS system, Model 5 (Perkin Elmer, Shelton, CT, USA) was used coupled with GC HP 5972 (Agilent Technologies, Palo Alto, CA, USA), equipped with a HP‐5 capillary column. Oven temperature was programmed from 45 to 240 °C at 3 °C/min; injector temperature was fixed at 250 °C; carrier gas, helium (0.5 ml/min). The MS operating parameters were interface and ion source temperatures were 300 °C, 200 °C respectively, EI mode: 70 eV, scan range: 40–400 amu.

Compounds identification was established through mass spectra of the individual GC peaks by a computer search of the commercial libraries (Wiley, NIST), as well as matching with the published mass spectra. The identification was further confirmed by the calculation of the retention indices (RI) relative to (C6–C22) n‐alkanes.

### Headspace solid-phase micro-extraction coupled with gas chromatography-mass spectrometry (HS-SPME)

The volatile scent of blooming flowers was trapped on SPME (solid phase micro extraction), which was desorbed at the injection port at 210 °C for 1 min. The analysis was performed using Shimadzu gas chromatograph (Model GC-17A) coupled with Shimadzu model QP-5000 mass spectrometer (Tokyo, Japan) according to Farag et al., 2017^49^. The identification of volatile components was established using AMDIS software (https://www.amdis.net) along with their retention indices (RI) as compared to n-alkanes (C6–C20). In addition to, mass spectrum matching with NIST, WILEY library database using matching score more than 800.

### Green synthesis of MgO nanoparticles

The aqueous extract of the dried powder of *Rosa* flower (2% w/w in double distilled water) was heated at 80 °C for 40 min with vigorous stirring and filtered through Whatman’s No.1. Then it was subsequently mixed with 0.1 M solution of magnesium nitrate hexahydrate Mg(NO_3_)_2_·6H_2_O in the proportion of 10: 90 w/w with continuous stirring for 6 h at 90 °C and left to settle for 24 h. at room temp (25 °C). The solid–liquid dispersion was centrifuge at 10,000 rpm for 10 min. Finally, MgONps pellets were washed twice with distilled water and carefully collected, sonicated at 40 °C for 1 h and dried in hot air oven at 90 °C for 2 h^29^. MgONps were subjected to various analyses to confirm the nanoparticle formation.

### Spectrophotometric characterization of MgO nanoparticles

The synthesized of MgONps of *Rosa* flowers (RcNps) was monitored after precipitate formation using UV–Visible spectrophotometer (Milton-Roy Spectronic 1201), at a wavelength range 200–600 nm. The morphological features were analyzed using Field Emission Scanning Electron Microscope SEM Model Quanta 250 field emission gun (FEG) attached with EDX Unit (energy dispersive x-ray analyses), with accelerating voltage 30 kV, magnification × 14 up to 1,000,000 and resolution for Gun.1n), FEI company, Netherlands;the sample was coated with gold before analysis with SEM.

While the size of RcNps was characterized using High Resolution Transmission Electron Microscope HR-TEM, (JEOL GEM-2100, Tokyo, Japan), operated with high tension value 200 kV. The morphology of the resulting NP was analyzed immediately after preparation. The functional groups attached to the surface of nanoparticles and the other surface chemical residues were detected using FTIR by using the spectral range 4000–400 cm^−1^ with the resolution of 4 cm^−1^. The crystalline phase of the synthesized nanoparticles was analyzed by X-ray diffractometer (D8 Advance, Bruker, Germany). Data were recorded using Cu-Kα radiation (λ = 1.54 Å), in the 2θ range from 3° to 70° with an accelerating voltage of 40 kV.

X- ray photoelectron spectroscopy (XPS) was employed to to investigate the surface elemental composition using Thermo Scientific K-ALPHA (K-Alpha photoelectron spectrometer, Thermo Fisher Scientific Inc, USA) coupled with monochromatic X-ray ALK-alpha radiation (− 10 to 1350 eV) as a source of ionizing radiation.

### In-vitro assessment of RcNps as antioxidant

#### Superoxide anion scavenging activity

Superoxide anion scavenging activity was assayed as described by Nishikimi et al., 1972^50^ with minor modifications. The modified method based on the generation of superoxide radicals by non-enzymatic phenazine methosulfate (PMS) / nicotinamide adenine dinucleotide (NADH) system that reduces nitro blue tetrazolium (NBT) to a purple formazan under aerobic conditions. The reaction mixture contained phosphate buffer (20 mM, pH 7.4), PMS (60 μM), NADH (468 μM), NBT (156 μM), and various concentrations (0–100 μg/ml) of the sample or ascorbic acid, incubated at room temperature for 5 min, and measured at 560 nm using UV-vis spectrophotometer. A control reaction contains all reagents without the tested samples. The radical scavenged percent was calculated using the following formula:

1 $$\;Radical\;Scavenged\;\;\% \; = \;\frac{Control\;absorbance\;\,\;\,Test\;absorbance\;}{{Control\;absorbance}}\; \times \;100$$

### Nitric oxide scavenging activity

Nitric oxide radical scavenging assay based on Griess Illosvoy reaction reported by Atere et al., 2018^51^ with slight modifications. Briefly, the reaction mixture in a final volume of 1 ml containing sodium nitroprusside (0.9 ml, 10 mM) in phosphate buffer saline (pH 7.4) and tested extracts or ascorbic acid at different concentrations (0–100 μg/ml, 0.1 ml) was incubated at 25 °C for 150 min. After incubation, the reaction mixture was mixed with 1% sulphanilamide (0.5 ml, in 5% phosphoric acid) and incubated in the dark for 10 min. Then, naphthyl ethylene diamine di-hydrochloride (0.5 ml, 0.1% w/v) was added, and allowed to stand for 30 min at 25 °C. The absorbance of the pink colored solutions was measured at 540 nm against blank solutions. The radical scavenged percent was calculated according to formula (1).

### Hydroxyl radical scavenging activity (oxidative degeneration of deoxyribose)

Hydroxyl radical scavenging activity was carried out as described by Halliwell et al., 1987^52^ with minor modifications. Hydroxyl radical (OH^•^) was generated by Fenton reaction (Fe^3+^ ascorbate-EDTA-H_2_O_2_ system). The reaction mixture in a final volume of 1 ml contained 2-deoxyribose (10 mM), phosphate buffer (20 mM, pH 7.4), FeCl_3_ (10 μM); ethylene diamine tetracetic acid (EDTA, 100 μM), hydrogen peroxide (H_2_O_2_, 10 mM), ascorbic acid (100 μM) and various concentrations of the tested samples or reference compound. After incubation for 1 h at 37 °C, 0.5 ml of the reaction mixture was added to 1 ml of 10% trichloroacetic acid (TCA), followed by addition of 1 ml of 1% thiobarbituric acid (TBA). Then, the mixture was incubated at 90 °C for 60 min to develop the pink color. After cooling, the absorbance was measured at 532 nm. The radical scavenged percent was calculated according to formula (1).

### Inhibition of xanthine oxidase

Xanthine oxidase (EC 1.1.3.22) activity was determined by monitoring the release of uric acid from xanthine according to Sigma protocol with minor modifications. The reaction mixture containing varying concentrations (0-100 μl/ml) of tested samples (100 μl) mixed with xanthine oxidase (100 μl, 0.2 U/ml) and potassium phosphate buffer (300 μl, 50 mM, and pH 7.4) and water (100 μl) was incubated at 37 °C for 15 min. Then xanthine (200 μl, 0.15 mM) as a substrate was added to each sample and further incubated at 37 °C for 30 min, the change in absorbance was measured at 295 nm. Inhibition of xanthine oxidase activity was expressed as the percentage and was calculated using the following formula:

2 $${\text{Enzyme inhibition }}\% \, = \, \left[ {{1 } - \, \left( {{\text{Enzyme activity in the presence of test extract }}/{\text{ activity without test extract}}} \right)} \right] \, \times { 1}00$$

### In-vitro evaluation of RcNps as anti-aging

The anti-aging potential of the tested samples were assessed by their ability to inhibit collagenase, elastase, hyaluronidase, and tyrosinase enzyme activities using epigallocatechin gallate (EGCG) as reference agent^53,54^. The assay in the absence of any enzyme was considered as control value for maximum inhibition. All the reactions were performed in triplicates. Inhibition of enzyme activity was expressed as the percentage (formula 2).

### Anti-collagenase activity

Inhibition of collagenase (EC 3.4.24.3) activity was performed by the Spectro-photometric method highlighted by Van Wart & Steinbrink, 1981^55^ with minor modifications. The reaction mixture contained Tricine buffer (50 μl of 50 mM and pH 7.5), tested samples (25 μl) at different concentrations (0–100 μg/ml) and Clostridium histolyticum collagenase enzyme (25 μl, 0.8 U/ml) was incubated for 15 min. Synthetic substrate 2-furanacryloyl-l-leucylglycyl-l-prolyl-l-alanine (FALGPA, 50 μl of 2 mM) was added. The change in absorbance was recorded immediately at 340 nm for 5 min using micro plate reader (BioTEk Instruments Inc., USA). Then ΔA340/min was calculated.

### Anti-elastase activity

Inhibition of elastase (EC 3.4.21.36) activity was evaluated by a spectrophotometric method^56^ with slight modifications. Briefly, the test samples (50 μl) at different concentrations (0–100 μg/ml) and Tris-HCl buffer (150 µL of 0.1M, pH 8.0) were incubated with the porcine pancreatic elastase (25 μl of 0.03 U/ml) at 25 °C for 15 min. The reaction was started with the addition of *N*-Succinyl-Ala–Ala–Ala-*p*-nitroanilide as a substrate (25 μl of 1 mM in Tris-HCl buffer). The change in absorbance recorded directly at 410 nm for 5 min using micro plate reader (BioTEk Instruments Inc., USA). Then ΔA410/min was calculated.

### Anti-hyaluronidase

Inhibition of hyaluronidase (EC 3.2.1.35) was performed following Sigma protocol with slight modifications. The reaction mixture contained hyaluronidase (100 µL of 4 U/ml), sodium phosphate buffer (100 µl of 200 mM, pH 7, 37 °C), sodium chloride (77 mM) and Bovine Serum Albumin, BSA (0.01%), and different concentration of sample solution (25 µl) was incubated at 37 °C for 10 min. Then, the reaction was initiated by the addition of hyaluronic acid (100 µl of 0.03% in 300 mM sodium phosphate, pH 5.35) as a substrate and re-incubated at 37 °C for 45 min. Acid albumin solution (1 ml of 0.1% BSA in 24 mM sodium acetate and 79 mM acetic acid, pH 3.75) was added to precipitate the undigested hyaluronic acid. This mixture was left at room temperature for 10 min and centrifuged. The absorbance of the supernatant was measured at 600 nm using Unicom spectrophotometer.

### Anti-tyrosinase

Inhibition of tyrosinase (EC 1.14.18.1) was established as described by Sigma protocol and Nguyen et al., 2016^57^. Briefly, tested samples (1 ml) at different concentration and mushroom tyrosinase (100 µl of 15 U/ml) were mixed in phosphate buffer (0.9 ml of 0.1 mM, pH 6.8) and pre-incubated for 30 min at 25 °C. The enzyme substrate, l-DOPA solution (1 ml of 1.5 mM in 0.1 M phosphate buffer pH 6.8) was added to initiate the reaction. After incubation at 25 °C for 10 min, the absorbance was recoded at 475 nm with Unicom spectrophotometer.

### Antibacterial assay against biofilm forming bacteria

MICs were determined for all the tested bacterial strains according to the guidelines of the Clinical and Laboratory Standards Institute^58^. Briefly, the bacterial suspensions were prepared by suspending bacterial cultures in sterile normal saline for 18 h. The turbidity of the bacterial suspensions was adjusted to 0.5using McFarland standard (equivalent to 1.5 × 10^8^ CFU/ml). RcNps were prepared in Milli-Q water and diluted with twofold serial dilutions with Mueller–Hinton Broth in concentration (1.95 to 1000 µg/ml). While, the bacterial suspension was diluted with MHB (5 × 10^5^ CFU/ml per well). The plates were incubated at 37 °C for 18 h and visually observed for the absence or presence of turbidity. Similarly, MICs of ciprofloxacin were recorded.

### Biofilm susceptibility assay and determination of minimum biofilm inhibitory concentrations

The biofilms of selected strains were prepared in 96-well flat-bottom polystyrene microtiter plates using a previously described method^59^ with minor modification, after the biofilms formation, crystal violet staining, and quantification of biofilms as previously described ,the percentage of biofilm inhibition was calculated according to the formula:

$$1-\frac{\mathrm{ODs}}{\mathrm{ODc}}\times 100{\%}$$

where, ODs is the mean optical density of wells treated with the samples and ODc is the mean optical density of untreated wells. The relation between the percentages of biofilm inhibition and the sample concentrations is plotted to get biofilm inhibitory curves. The minimum biofilm inhibitory concentration (MBIC) is the lowest concentration required to completely inhibit biofilm formation. The absorbance was observed at 595 nm using a microplate reader (Spectra easy microplate reader; Inverness Medicals, India).

### Statistical analysis

Data is represented as mean ± standard error (SE). The inhibitory activity of the tested samples was expressed as IC_50_ and compared with that of reference agent. All tests were performed in triplicate and the data expressed as the mean of three parallel measurements (n = 3). The differences between means were statistically tested by One-way ANOVA followed by Dunnett's multiple comparisons test. Significant difference from corresponding reference standard was tested at *P* < 0.05, 0.01 and 0.001.

## Conclusion

To the best of our knowledge, this study represents the first comprehensive investigation utilizing the green synthesis of MgO nanoparticles loaded with *Rosa floribunda charisma* with highlights of its volatile aroma extracted by both traditional and advanced methods. Phenyl ethyl alcohol was the most prominent component with the unique rose scent. MgONps were characterized using different spectroscopic method viz; UV–Visible (UV–Vis) Spectroscopy, FTIR, HR-TEM, FE-SEM, EDX, XRD, and XPS. Moreover, MgONps exhibited a promising antioxidant and antiaging activities in dose dependent manner. In addition to, its potential antibiofilm activity against skin born bacteria; *S. epidermdis*, *S. pyogenes* and *Pseudomonas aeruginosa*. Outcome of results revealed that the synthesized nanoparticles utilizing *Rosa floribunda charisma* may be considered as a new leading natural source of antibacterial and antiaging agent in the field of skincare cosmetic industry.

## Supplementary Information

Supplementary Figures.

## Abbreviations

EDS: Energy dispersive analysis of X-ray
ECM: Extracellular matrix
EGCG: Epigallocatechin gallate
EDX: Energy dispersive X-ray spectroscopy
FTIR: Fourier transform infrared spectroscopy
FFT: Fast fourier transform of high resolutiontransmission electron microscope
FRTA: Free radical theory of aging
GC–FID: Gas chromatography coupled with flame ionization detectors
MAE: Microwave assisted extraction
MBIC: Minimum biofilm inhibitory concentration
MHB: Mueller–Hinton broth
MIC: Minimum inhibitory concentration
MgONps: Magnesium oxide nanoparticles
NADH: Nicotinamide adenine dinucleotide
NBT: Nitro blue tetrazolium
NO: Nitric oxide
RcNps: Nano form of *Rosa floribunda*
ROS: Reactive oxygen species
PMS: Phenazine methosulfate
TBA: Thiobarbituric acid
TCA: Trichloroacetic acid
HR-TEM: High resolutiontransmission electron microscope
SEM: Scanning electron microscope
SPME: Solid phase microextraction
XPS: X-ray photoelectron spectroscopy
XRD: X-ray diffraction
