# Supplementary material for: Green synthesis of magnesium nanoparticles mediated from Rosa floribunda charisma extract and its antioxidant, antiaging and antibiofilm activities
Source: Sci Rep. 2021 Aug 19;11:16868. doi: 10.1038/s41598-021-96377-6 (PMC8376960; doi:10.1038/s41598-021-96377-6)
Supplement: Supplementary file 1 — Supplementary Figures. [file 41598_2021_96377_MOESM1_ESM.docx]

Green synthesis of magnesium nanoparticles mediated from *Rosa* *Floribunda* charisma extract and its antioxidant, antiaging and antibiofilm activities

Inas Y. Younis ^a^*, Seham S. El-Hawary ^a^, Omayma A. Eldahshan ^b^, Marwa M. Abdel-Aziz ^c^, Zeinab Y. Ali ^d^

^a^ Department of Pharmacognosy, Faculty of Pharmacy, Cairo University, Cairo 11562, Egypt.

^b^ Pharmacognosy Department, Faculty of Pharmacy, Ain Shams University, Cairo, Egypt.

^c^ Medical Microbiology at the Regional Center for Mycology and Biotechnology, Al-Azhar University, Cairo, Egypt.

^d^ Department of Biochemistry, National Organization for Drug Control and Research (NODCAR), 12553 Giza, Egypt.

**Supp 1.** The main classes of volatile compounds extracted from *R. floribunda* charisma by three different methods.

**
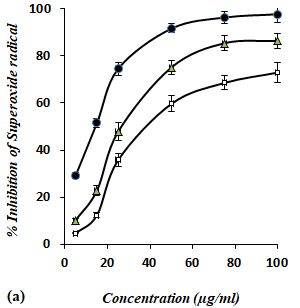

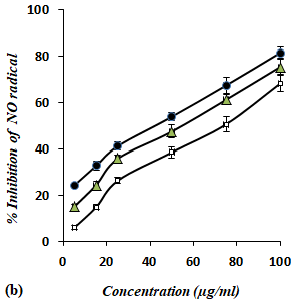
**

**
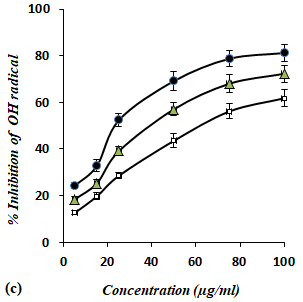

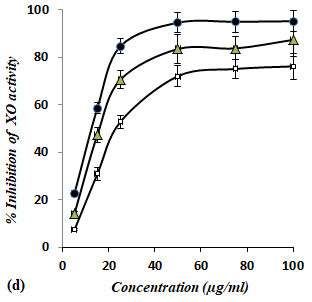
**

**
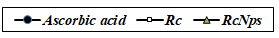
**

**Supp2.** Dose-response curve of antioxidant activity of the extract of *Rosa* (RC) and its nano-form (RcNps) in comparable to ascorbic acid. (a-c): % inhibition of superoxide, nitric oxide and hydroxyl radicals, respectively; (d): inhibition of xanthine oxidase. The data represent the mean of % of three replications ± S.E.

**
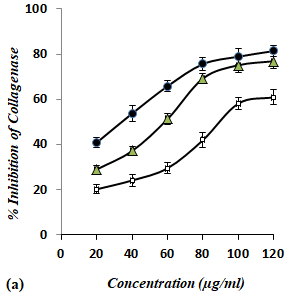

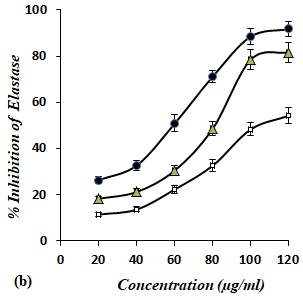
**

**
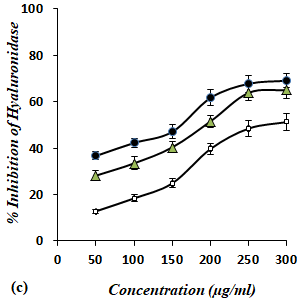

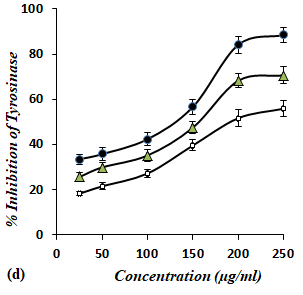
**

**
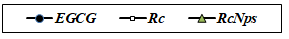
**

**Supp3.** Dose-response curve of anti-aging activity of the extract of *Rosa* (RC) and its nano-form (RcNps) in comparable to epigallocatechin-3-gallate (EGCG). The data represent the mean of three replicates ± S.E.
